# Factors associated with depression during pregnancy in women receiving high- and low-risk prenatal care: a predictive model

**DOI:** 10.3389/fpsyt.2024.1326151

**Published:** 2024-07-09

**Authors:** Jessica Gorrão Lopes Albertini, Glaucia Rossana Guerra Benute, Rossana Pulcineli Vieira Francisco, Marco Aurélio Knippel Galletta

**Affiliations:** ^1^ Disciplina de Obstetricia, Departamento de Obstetricia e Ginecologia da Faculdade de Medicina FMUSP, Universidade de Sao Paulo, Sao Paulo, Brazil; ^2^ Coordination of the Psychology Course, Sao Camilo University Center, Sao Paulo, Brazil

**Keywords:** depression, pregnancy, high-risk pregnancy, epilepsy, diabetes, epidemiological models

## Abstract

**Introduction:**

Depression during pregnancy can put strain on pregnant women’s interpersonal relationships, the formation of emotional bonds with the fetus, and the adaptation to the new routine and social role post-pregnancy. Some studies have associated socioeconomic factors, emotional factors, interpersonal relationships, perceived social support, gestational risk, and the occurrence of certain diseases during pregnancy with higher risk of depression.

**Objectives:**

This study aimed to investigate the prevalence of depression during pregnancy and associated factors in low- and high-risk prenatal patients at a Brazilian university hospital.

**Methods:**

This study presents a retrospective and prospective cross-sectional design. A total of 684 prenatal psychological analysis records from a Brazilian tertiary university service were retrospectively evaluated to assess depression through the PRIME-MD questionnaire between 2002–2017. Between 2017 and 2018, 76 patients treated at the same service were prospectively evaluated with the aforementioned instrument. Medical records were accessed to obtain labor and birth data. Multivariate analyses assessed the association between sociodemographic, gestational or obstetric, and health variables and the presence of depression during pregnancy.

**Results:**

A total of 760 pregnant women were included in the study, with a depression prevalence of 20.66% (n = 157). At the time of assessment, 48 (21.05%) women from the low-risk pregnancy group and 109 (20.49%) from the high-risk pregnancy group were depressed. The mean age was 30.01 ± 6.55 years in the group with depression and 29.81 ± 6.50 years in the group without depression. In the univariate analysis, there was an association of risk for depression with absence of paid work, absence of a partner, low family income and diagnosis of epilepsy, being a protective factor the presence of diabetes during pregnancy. However, in the multivariate analysis, a lower family income, not having a partner at the time of the assessment, and the prevalence of epilepsy were independently associated with an increased risk of depression during pregnancy.

**Conclusion:**

This study showed that 1 in 5 women had depression during pregnancy, with no association with obstetric risk, but those women living in unfavorable economic conditions, without a partner, and having epilepsy were at increased risk of depression.

## 1 Introduction

Pregnancy is a normal physiological phenomenon and a significant life experience for many. Within this context, some women may experience the expected transformations for this condition, meaning that maternal and fetal risk is within the average identified in the general population, resulting in a low-risk pregnancy, while others may have a higher risk for unfavorable outcomes, thus constituting a high-risk pregnancy (1). Factors associated with high-risk pregnancy include individual characteristics, maternal diseases, and unfavorable socioeconomic conditions (2).

Both low- and high-risk pregnancies present several physical, hormonal, psychological, and social integration changes that lead to intense transformations and can affect the woman’s mental health in ways still requiring further studies to be fully understood (3–5). Depression is the most prevalent psychiatric disease during pregnancy. A common characteristic among depressive disorders is the presence of a sad or depressed mood, with a lack of interest or pleasure in practically all activities and feelings of guilt and low self-esteem (6, 7).

The prevalence of depression during pregnancy ranges between 9% (8) and 61.4% (9), with lower indices in low-risk pregnancies (8, 10) and higher indices in high-risk pregnancy (8, 9). These percentages may vary depending on the geographic location, level of development of each country, method used in the study, and the instrument used to assess depressive symptoms (11). Data from a meta-analysis study (7) published in 2021 estimate that the mean global prevalence of depression during pregnancy is 20.7% (95% CI 19.4–21.9%), reducing to 15% when only major depression is considered, thus constituting one of the most common clinical complications in pregnancy.

Considering the peculiarities of depressive symptoms and their repercussions on the pregnant woman’s daily life, for example, on her behavior, self-perception, and understanding of pregnancy, a growing number of studies (3, 12, 13) have investigated the possible impacts of depression during pregnancy, uggesting that it would lead to worse obstetric results and unfavorable neonatal outcomes.

Several authors have studied risk factors for depression during pregnancy, classifying them as biological and psychosocial risks (14) that include mainly a history of mental disorder (15, 16), history of domestic violence or abuse (17, 18), lack of social support (16, 19, 20), unplanned current pregnancy (21), smoking, and history of miscarriage (22).

The influence of socioeconomic aspects seems to be quite significant. Previous systematic review and meta-analysis studies, such as that by Nisar et al. (20), associated lower socioeconomic levels with depression in Chinese pregnant women. In particular, higher education levels and better living conditions were protective factors. Simultaneously, other meta-analyses (7) demonstrated that unemployment is also associated with depression during pregnancy and that greater attention should be given to this group, particularly in low- and middle-income countries, because prevalence data is higher in these geo-economic conditions.

However, few studies on factors predisposing to gestational depression have been conducted in Latin America. Guidelines and recommendations from European (23), North American (24), and Canadian (25) organizations and societies suggest screening for depressive symptoms during prenatal care as an important opportunity for the early identification and treatment of signs and symptoms of depression during pregnancy. The most commonly used standardized tool for this purpose is the Edinburgh Postnatal Depression Scale (EPDS), a self-report screening instrument used with various cutoff scores to detect signs of risk or a high probability of a major depressive disorder diagnosis. However, such results are more precisely applicable to the postnatal period, and a review on this topic (26) indicated that, in the assessment of pregnant women, the EPDS result should be associated with a clinical evaluation by a specialized professional. Building on this premise, the use of the PRIME-MD in evaluating depressive symptoms in pregnant women becomes a more favorable possibility as it contains within its structure the DSM-III diagnostic criteria for major depressive disorder, serving as a guide for specialized clinical interviews.

Understanding the influence of depression in pregnancy and the possible associated factors in 102 specific populations are relevant to improving the care provided to these women, helping reduce 103 deleterious effects on the mother-baby dyad (22). There are few publications in Latin 104 America, especially in Brazil, exploring factors related to antenatal depression, and even fewer 105 exploring depression in populations at high obstetric risk (27–29). Therefore, this study aimed to investigate the prevalence of depression 108 during pregnancy and associated factors in low- and high-risk prenatal patients in a Brazilian 109 university hospital.

## 2 Methods

A time series study with retrospective and prospective data between 2002 and 2018 was 112 conducted in a public tertiary university hospital in São Paulo, SP, Brazil, with the project previously 113 approved by the institution’s Research Ethics Committee under number 68144317.2.0000.0068. As of June 2017, a psychological screening service was implemented to identify women who needed specialized mental health care among patients starting prenatal care. The care protocol used in the psychological screening service also consisted of an initial semi-structured interview and the PRIME-MD depression module.

**Figure 1:**
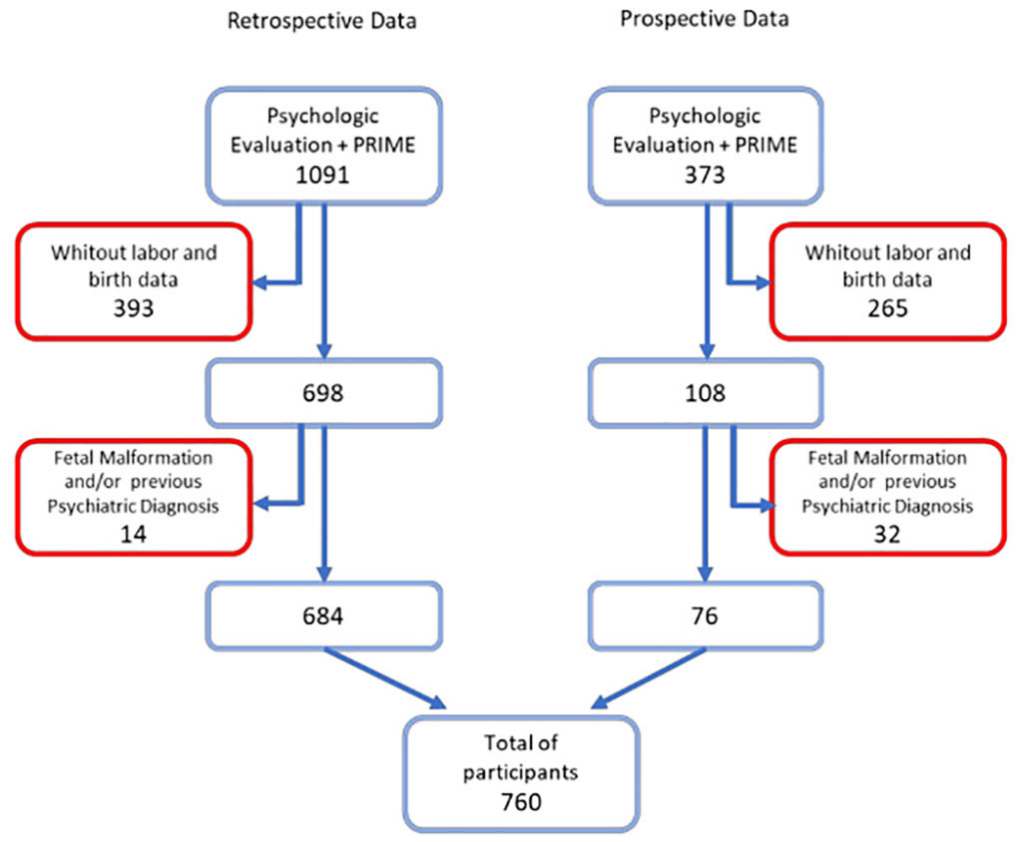
Data collection flow chart. Embedded text: Retrospective data; PRIME-MD psychological evaluation; No labor and birth data; Fetal malformation or previous psychiatric diagnosis and the total number of participants; Prospective data.

The study included all patients evaluated by the PRIME-MD depression module between January 2002–June 2018, including both phases. The exclusion criteria were: not completing the interview, not completing the depression assessment via PRIME-MD, having received a fetal malformation diagnosis in the current pregnancy, and reporting a prior diagnosis of mood disorder or other mental disorders. Therefore, of 1,091 psychological records containing depression evaluation by the PRIME-MD identified in the retrospective part of the research, 407 were excluded, leaving 684 pregnant women in the study. In the prospective part, 373 depression evaluations were conducted in psychological screening, with 297 cases excluded and 76 pregnant women included. Therefore, a total of 760 pregnant women were included in the study. **Figure 1** shows the study flowchart.

Several demographic, social, clinical, and obstetric variables were surveyed to investigate a possible association with a diagnosis of depression by the PRIME-MD. The following demographic variables were recorded: age, education level, religion, and marital status. Furthermore, the participant’s personal and family per capita income were presented in minimum salaries corresponding to the period during which the pregnant woman was evaluated (minimum salary, MS, in 2018 was BRL 954, i.e., around USD 3,000). The following obstetric variables were analyzed: parity, previous miscarriages, complications, and gestational age at the time of psychological evaluation. A high risk pregnancy was considered when there was a risk of death or morbidity both for the mother and fetus, including intercurrent clinical pathologies (hypertension, diabetes, heart disease, thyroid disease, infectious diseases, thrombophilia, neoplasms, asthma, epilepsy, collagenosis) or obstetric history of risk. The remaining pregnant women were included in the low obstetric-risk group.

The Primary Care Evaluation of Mental Disorders - PRIME-MD is an instrument composed of five modules (mood, anxiety, eating, somatoform disorders, and alcohol abuse or dependence disorders), which can be used independently. The PRIME-MD mood module’s main objective is identifying whether the patient is in a major depressive episode, as it contains the criteria established by the DSM for this diagnosis. The Portuguese version, revised by Dr. Robert Leopold Spitzer, its creator, has demonstrated good accuracy (sensitivity and specificity) for major diagnostic groups in primary care settings. For the present study, the module that evaluates mood disorders, specifically Major Depression, was used (30, 31) and considered positive for depression when at least five out of nine symptoms evaluated by the PRIME-MD were present, of which it must necessarily include the presence of sad and depressed mood or lack of interest/pleasure in situations previously experienced as pleasurable.

Qualitative variable results were presented as relative (percentage) and absolute (n) frequencies.

Quantitative variables were presented as the mean, median, minimum, and maximum values and standard deviation.

Distribution tests were conducted for quantitative variables to verify data normality. Quantitative variables were compared between groups using the Student’s t-test for parametric variables or the Mann–Whitney test for non-parametric variables. The Kruskal-Wallis non-parametric test was used to reach more than two groups.

Pearson’s chi-square or Fisher’s exact tests assessed the association between two categorical variables. The correlation coefficient between two quantitative variables was evaluated by Pearson’s statistical test or, in the case of non-normality, by Spearman’s statistical test.

The association of these variables with depression during pregnancy was demonstrated by odds ratio with CI95%, crude or adjusted to the predictive model. A predictive model for depression was used to analyze risk factors independently associated with depression during pregnancy, which was constructed with the variables most significant in univariate analysis. This analysis used a logistic regression model of characterization variables and health data with p < 0.200, through the backward stepwise model. Regression model adequacy was verified using the Hosmer-Lemeshow test.

The significance level was set at 5%—results with a p-value less than 0.05 were considered significant. The IBM SPSS software version 20. was used for data analyses.

## 3 Results

Between 2002 and 2017, pregnant women undergoing outpatient prenatal care referred for psychological assessment and those receiving face-to-face psychological care were included in an initial care protocol consisting of a semi-structured interview and assessment of mood using the major depressive disorder module of the Portuguese version of the Primary Care Evaluation of Mental Disorders (PRIME-MD) (30, 31), and these assessments were attached to the patient’s medical records. The retrospective cohort was generated through the review of evaluations carried out with the same instrument in cases referred by OG doctors, who provide prenatal care to these patients, for Psychology evaluation. The prospective cohort consisted of the psychological screening assessment of pregnant women at the time of the first prenatal consultation, without prior medical referral.

The total prevalence of depression in the study sample (n = 760) was 20.66% (n = 157). This rate did not consider if the pregnant woman was in the low (21.1%) or high (20.5%) obstetric risk group. Considering only the population diagnosed with depression (n = 157; 100%), 84.71% (n = 133) were in the high-risk group.

**Table 1** presents the study population characterization data, variables evaluated, the association with depression during pregnancy, and the ORcrude and confidence intervals. There was no statistically significant difference between pregnant women with or without depression symptoms when considering the associated gestational risk (p= 0.492).

**Table 1:** Distribution of characterization, sociodemographic, and health data in mean, minimum and maximum values; relative and absolute frequencies; ORcrude (CI95%); and symptoms associated with depression during pregnancy.

Sociodemographic and pregnancy data	Depression		*p*	ORcrude (CI95%)
	Yes	No		
Age				
Median (Min–Max)	31 (18-45)	30 (18-45)	0.721**	0.996 (0.976-1.016)
Personal income - minimum salaries				
Up to 1 minimum salary/month	7/25 (28.0)	18/25 (72.0)	0.092***	Ref.
Between 1 and 5 minimum salaries/month	48/266 (18.0)	218/266 (82.0)		0.566 (0.224-1.431)
Between 6 and 10 minimum salaries/month	4/13 (30.8)	9/13 (69.2)		1.143 (0.224-4.951)
No income	63/240 (26.3)	177/240 (73.8)		0.915 (0.365-2.295)
Family Income—Per capita minimum salaries				
≥ 2 minimum salaries/month	80/458 (17.5)	378/458 (82.5)	0.002***	Ref.
< 2 minimum salaries/month	59/209 (28.2)	150/209 (71.8)		1.858 (1.264-2.734)
Gestational age at the time of evaluation				
Median (Min–Max)	24/49 (7-40)	25/49 (4-40)	0.597**	0.996 (0.976-1.016)
Gestational age at the time of evaluation				
First trimester	12/76 (15.8)	64/76 (84.2)	0.183	Ref.
Second trimester	83/354 (23.4)	271/354 (76.6)		1.633 (0.841-3.172)
Third trimester	58/308 (18.8)	250/308 (81.2)		1.237 (0.627-2.441)
Age				
< 20	9/47 (19.1)	38/47 (80.9)	0.953	Ref.
20 a 35	113/548 (20.6)	435/548 (79.4)		1.097 (0.515-2.6335)
> 35	35/165 (21.2)	130/165 (78.8)		1.137 (0.502-2.573)
Schooling				
Up to 5 years (elementary school)	80/333 (24.0)	253/333 (76.0)	0.140	Ref.
Up to 12 years (high school)	55/299 (18,4)	244/299 (81.6)		0.713 (0.485-1.048)
Over 12 years (university)	22/125 (17.6)	103/125 (82.4)		0.675 (0.400-1.141)
Relationship status (with partner)				
With partner	129/656 (19.7)	527/656 (80.3)	**0.029**	1.761 (1.061-2.923)
Without partner	25/83 (30.1)	58/83 (69.9)		
Paid activity				
Yes	74/403 (18.4)	329/403 (81.6)	**0.037**	1.469 (1.023-2.109)
No	76/306 (24.8)	230/306 (75.2)		
**First pregnancy**	46/262 (17.6)	216/262 (82.4)	0.126	0.742 (0.507-1.088)
**Nulliparous woman**	61/343 (17.8)	282/343 (82.2)	0.076	0.723 (0.505-1.035)
High-risk pregnancy				
Yes	133/629 (21.1)	496/629 (78.9)	0.92*	1.184 (0.731-1.919)
No	24/130 (18.5)	106/130 (81.5)		
**Previous Abortion (yes)**	49/223 (22.0)	174/223 (78.0)	0.564	1.119 (0.764-1.637)
**Religion (yes)**	103/517 (19.9)	414/517 (80.1)	0.198*	1.294 (0.873-1.917)
**Complications in previous pregnancies**	54/269 (20.1)	215/269 (79.9)	0.198*	1.319 (0.865-2.014)
**Diseases in current pregnancy**	132/623 (21.2)	491/623 (78.8)	0.464*	8.838 (0.521-1.347)
**Diabetes**	39/240 (16.3)	201/240 (83.8)	0.040***	0.659 (0.442-0.983)
**Hypertension**	35/170 (20.6)	135/170 (79.4)	0.980*	0.995 (0.652-1.516)
**Cardiopathy**	10/59 (16.9)	49/59 (83.1)	0.464*	0.769 (0.380-1.555)
**Thyroid diseases**	10/50 (20.0)	40/50 (80.0)	0.905*	0.957 (0.468-1.960)
**Infectious diseases**	10/30 (33.3)	20/30 (66.7)	0.080*	1.983 (0.909-4.327)
**Thrombophilia**	7/30 (23.3)	23/30 (76.7)	0.715*	1.175 (0.495-2.790)
**Neoplasms**	7/29 (24.1)	22/29 (75.9)	0.643***	1.230 (0.516-2.934)
**Asthma**	6/17 (35.3)	11/17 (64.7)	0.136**	2.138 (0.778-5.875)
**Epilepsy**	5/8 (62.5)	3/8 (37.5)	0.012**	6.579 (1.555-27.834)
**Collagenosis**	3/24 (12.5)	21/24 (87.5)	0.444**	0.539 (0.159-1.830)
**Repeat miscarriage**	3/18 (16.7)	15/18 (83.3)	0.472**	0.764 (0.218-2.671)

HC-FMUSP, 2002–2018. *Student’s t-test; **Mann-Whitney test; ***Chi-square test; ****Fisher’s exact test.

The bold values refer to p-values less than 0.05.

The analyses indicated a statistically significant association between per capita family income in minimum wages and depression, where the majority of participants in the depression group had income lower than 2 minimum salaries/month (p = 0.002). The pregnant women who reported not engaging in paid work had a higher risk of belonging to the depression group (OR 1.469; CI95% 1.023–2.109). Similarly, not having a partner showed a statistically significant association with depression, with greater risk for participants without a partner at the time of the evaluation (OR 1.761; CI95% 1.061–2.923). Regarding clinical variables, there was a significant statistical association between depression and the diagnosis of diabetes and epilepsy. The group without diabetes showed a greater chance of depression during pregnancy than the group with diabetes (OR 0.659; CI95% 0.442–0.983). Having epilepsy was associated with a greater risk of depression during pregnancy (OR 6.579; CI95% 1.555– 27.834).

Variables considered clinically relevant vis-à-vis depressive symptoms during pregnancy were also included. A stepwise backward model with 11 stages was included, maintaining the variables “religion,” “nulliparous,” and “diabetes” to adjust the model (Hosmer-Lemeshow = 0.685). The results of this analysis with the respective ORadjusted are presented in **Table 2**.

**Table 2:** Logistic regression model for the association between significant variables and depression during pregnancy adjusted for the control variables religion, being nulliparous, and having diabetes.

	*B*	*P*	*OR*	(CI95%)
Without partner	0.622	**0.036**	1.863	(1.041-3.334)
Epilepsy	1.560	**0.042**	4.758	(1.055-21.455)
Family incomes < 2 MS	0.577	**0.005**	1.780	(1.193-2.658)

HC-FMUSP, 2002–2018.

The bold values refer to p-values less than 0.05.

Thus, the final predictive model analysis showed that the chance of having depression in the group of pregnant women without a partner was 1.86-fold higher (CI95% 1.041–3.334). Prevalence of epilepsy was also an independent factor, with more than four-fold more significant chance of depression during pregnancy (OR = 4.758, CI95% 1.055–21.455). The variable family income < 2 MS, related to a worse socioeconomic condition, also maintained statistical significance in the predictive model com 1.78-fold increased chance of a participant this group having depression during pregnancy.

## 4 Discussion

The general prevalence of major depression in the sample was 20.66%. The scientific literature (8, 9) presents wide depression rate variation during pregnancy. However, the results of this study are comparable with other international studies (32, 33) that used assessment instruments constructed with the same theoretical assumptions (Structured Clinical Interview for DSM Disorders (SCID) or PRIME-MD). A North American study (32) using the SCID, an instrument used to diagnose depressive disorders, identified that 20% of pregnant women evaluated in the second trimester met the criteria for major depressive disorder. Rashid and Mohd (34) conducted a cross-sectional study with 3,000 Malaysian pregnant women undergoing prenatal care in all gestational trimesters. They identified a 20% prevalence of depressive symptoms, which corroborates ours findings. Still in this sense, a Korean study with 1,262 women assessed using the Edinburgh Postpartum Depression Scale (EPDS) identified that 20.2% of them scored above ten for depressive symptoms (35). Silva et al. (36) conducted a study in 2012 with 1,109 low-risk pregnant women in the second and third trimester of pregnancy reporting rates similar to ours, with the identification of depressive symptoms in 20.5% of the sample also using the EPDS, but with a cutoff point of 13.

Conversely, compared with the present study, some works (37, 38) with similar instruments presented lower prevalence results, indicating that different methodologies (such as inclusion or exclusion criteria and instrument evaluated) can explain, at least partially, different results. An important point is the chosen instrument for assessing depressive symptoms that can identify symptoms (symptom scales) or diagnose depressive disorder (diagnostic scales) and can focus decisively on the data obtained in studies and, consequently, on different rates of depression during pregnancy found in the literature. Specifically, there is an issue regarding using self-report instruments for symptomatological investigation, such as the EPDS, that identifies a more significant number of cases compared to clinical assessments using diagnostic instruments, such as the PRIME-MD. Juhas et al. (37) used the same instrument as in the present study (PRIME-MD). Nonetheless, they only had a sample of high-risk pregnant women, identifying that 11% of participants scored for depression, signaling the importance of specific group studies to discuss the specificity of each population. Similarly, the first longitudinal study in Latin America (8) to evaluate perinatal depression showed different findings compared to the present study, reporting lower depression rates. Their longitudinal study, when evaluating depressive symptoms using the Patient Health Questionnaire (PHQ-9), found that the percentage of participants exhibiting symptoms of depression was 16.6%. Another study (39) assessing major depression with the SCID showed a rate of 6.4%. Furthermore, other possible factors associated with depression prevalence variations in pregnant women include aspects related to different contexts of economic, social, cultural, and healthcare development of the studied population (7). One of the relevant aspects regarding the prevalence of depression was the choice of instrument, as it allowed a parameter based on the Diagnostic and Statistical Manual of Mental Disorders - DSM III-R (40) criteria, which could analyze pregnant women both at low and high gestational risk during all gestational trimesters.

As for data characterization, being without a partner was a significant difference factor in the univariate analysis between groups concerning depression, maintaining the association in the final predictive model with an even higher adjusted OR (ORadjusted = 1.863; 95% CI 1.041–3.334). Of the pregnant women with depression, around 30% reported not having a partner at the time of evaluation. Extensive literature (36, 41) on depression during pregnancy indicates relationship status as a critical associated factor. A study (42) with Dutch pregnant women identified a similar association between depression and not living with a partner at the beginning of pregnancy. However, this association was not maintained throughout pregnancy. Another study (43) with pregnant women treated at a university hospital in the city of São Paulo identified that participants with a partner had an 89% lower risk of depression during pregnancy.

An aspect related to the presence or absence of a partner indicated as necessary is the extent to which the relationship can be considered a protective factor. Studies such as the one by Redinger et al. (44) concluded that pregnant women reporting that their partners made life more difficult had a prevalence of depressive symptoms three-fold higher than the other women evaluated. These findings indicate that the gestational experience cannot be restricted only by women’s health. Instead, it needs to be expanded to consider their social support network and intimate relationships. Further, whether these relationships can be healthy, thus reducing or increasing the risk of mental disorders. In this scenario, the quality of the relationship and how this relationship can make the pregnancy experience difficult or easier should be assessed. One of the aspects of most significant clinical concern due to the potential risk to the maternal-fetal dyad is the presence of intimate partner violence, with physical, sexual, or psychological harm. Several studies (45–48) indicate that pregnant women exposed to threats or aggression have a greater chance of low adherence to prenatal care and of mental disorders, in particular, perinatal depression.

*Per capita* family income was another variable that showed a significant difference between groups, with the group with depression reporting lower mean family income than the group without depression, both in univariate (OR = 1.858; CI95% 1.264–2.734) and multivariate (OR 1.780; CI95% 1.193–2.658) analysis. This result corroborates other studies (49–51) reporting the impact of income, both of the pregnant women and the family, on the onset of depressive symptoms. A study by Choi et al. (49) indicated that unfavorable socioeconomic conditions were associated with a higher prevalence of depression in pregnant women. Similarly, Podvornik et al. (50) demonstrated an association between low socioeconomic status and depression during pregnancy in Slovenian women. Socioeconomic indicators and poverty measurement indices relate to a more significant presence of mental disorders. However, studies such as the one by Nasreen et al. (52) with women from Bangladesh identified no specific association with depression during pregnancy, diverging from our findings. Likewise, a study (53) with pregnant Indian women identified no statistical association between family income and depression during pregnancy.

The present study also indicated that having a paid job was a protective factor against the diagnosis of depression, with more than half of the participants in the group with depression stating that they had no paid work at the time of the evaluation. However, the association was not reflected in the multifactorial analysis of the predictive model. Meanwhile, studies such as the one by Algahtani et al. (54) demonstrated that unemployment increased the chance of depressive symptoms in Saudi women who had experience of paid work or were students. However, there is a lack of more extensive studies on pregnant women that consider individual aspects and broader socioeconomic contexts. During the study period, Brazil was experiencing an economic recession, with a significant gross domestic product (GDP) drop (55), unemployment rates of 12.2 million people, and unemployment levels of 11.6% of the population. Indexes from the last quarter of 2018 were twice as high as before the economic crisis in 2014 (55). This panorama may have influenced findings related to the sample characterization data in this study. It is essential to understand that depression during pregnancy is a multifactorial disorder requiring an approach and treatment that considers the patient’s social and economic aspects.

Of the maternal health variables analyzed in the present study, being in the group with a high-risk pregnancy, that is, having a disease associated with the current pregnancy, did not prove to be a risk factor for depression during pregnancy compared with a low-risk pregnancy. Unlike our findings, other studies (43, 56, 57) indicate that having a disease associated with pregnancy is a risk factor for depression during pregnancy. One of the most relevant aspects related to different results in the present study is the specific characterization of the group with low-risk women, mainly composed of women working in the institution’s own health department, which may generate bias.

Although the mental health of pregnant healthcare workers is an aspect little studied in the literature, it appears that there would be high rates of depression during pregnancy (58–60). One explanation would be a possible greater burden of physical and emotional stress which, associated with low social support, could lead to depression (61), but the impact of this association is yet to be determined, and there is still theoretical space for discussion about stress and its association with depression during pregnancy (62) Pregnant women exposed to stressors such as turbulent situations and psychological threats, who cannot adapt flexibly, appear to be at increased risk of depression during pregnancy.

On the other hand, we could think that such women, as they are often healthcare professionals, would be better able to take care of their own well-being, seeking to reduce work stress with alternative measures that encourage relaxation and a certain disconnection from problems. Such women would also have better knowledge of general guidelines and could better manage their health condition with better self-care conditions, which could improve their mental health condition. Therefore, which of these two trends would be the most prominent is something that needs to be further investigated, under different contexts.

Regarding diseases associated with the current pregnancy, diabetes was a statistically significant difference factor in univariate analysis; however, this difference did not remain significant in multivariate analysis as the presence of the variable improved the model, acting as an adjustment factor. The results of the present study indicated that being diabetic was a protective factor against depression during pregnancy, contradicting the literature (63–65) on the topic, which signals an association between these two variables. Notwithstanding, other authors could not establish an association between diabetes and depression (66, 67). The bidirectional relationship that may exist between depression and diabetes should also be noted. Studies with non- pregnant women indicate diabetes as a risk factor for depression (63, 66). However, depression is also a risk factor for the onset of diabetes (65). Furthermore, questions about the biological mechanisms underlying the two diseases could be shared (66). All the transformations and implications in a woman’s life form the hypothesis that diabetes, whether pre-existing or gestational, may increase the risk of depressive symptoms; however, evidence on the subject remains inconclusive.

We envision that diabetic patients in our sample could establish a secure positive bond with the multidisciplinary team, especially after following the guidance and implementing necessary lifestyle changes to achieve glycemic control with guidance and follow-up regarding diet control and monitoring of blood glucose levels through fingerstick tests. This could potentially reduce anxiety and stress, thereby lowering the risk of gestational depression. A study by Marquesim (66) at a university in São Paulo and another by Castro (67) at a university in Portugal reinforce this possibility. Even with the quality-of-life impairment and stress associated with this clinical condition, these patients presented depression rates equal to those of the general pregnant woman population. However, these conjectures and hypotheses require further studies to guide this specific group of pregnant women better.

Considering patients who reported having epilepsy, the univariate analysis identified, and the predictive model confirmed an increased risk of depression during pregnancy. We identified some studies that corroborate our findings. A study conducted in Norway (68) with 329 pregnant women with epilepsy and 106,224 without epilepsy reported a higher rate of depression in pregnant women with epilepsy than those without epilepsy.

Bjork et al. (69) identified higher rates of depressive symptoms in pregnant women with epilepsy, especially those who used more anticonvulsant medications. However, a recent prospective study (70) with pregnant women and women in the postpartum period compared women with epilepsy during pregnancy, pregnant women without epilepsy, and non-pregnant epileptic women. They observed no statistically significant difference between the three groups regarding the presence of depression diagnosed by the SCID. As discussed about the relationship between depression and diabetes, studies on epilepsy and depression point to a bidirectional association and indicate the need for a deeper analysis of this possible interaction. Some studies suggest that epilepsy and its resulting seizures induce frontal and temporal hypofunction, serotonergic and/or glutamatergic dysfunction, as well as hypofunction in the hypothalamic-pituitary-adrenal axis, processes that may be implicated in the occurrence of depressive symptoms. Although the present study did not assess the use of anticonvulsant medications, it is important to note that some studies (69, 70) indicate that their use may be associated with significant mood alterations and that polytherapies, epilepsy activity, and its severity appear to be bidirectionally related to the presence of psychiatric disorders. On the other hand, levetiracetam, which is known to cause behavioral changes (e.g., aggression, agitation, anger, anxiety, apathy, depression, hostility, and irritability), as well as psychotic symptoms, it only recently began to be used in our patients and was not present among the participants in the present study, during the period in which the research was carried. Furthermore, we can extrapolate and reflect on some possible risk aspects in these patients, such as loss of control, worsening of quality of life, and the potential increased number of seizures during pregnancy, requiring therapeutic regimen changes and leading to greater insecurity. These factors could increase depressive symptom rates.

The results of this study show that, in addition to classic risk factors associated with low family income and absence of a partner, epilepsy was found to increase the risk of depression, whereas diabetes was a factor that posed reduced risk. This study presents a relevant case series in a tertiary service where high- and low-risk obstetric patients are treated according to a well-established and safe protocols. These findings can guide similar services to increase attention to the profiles mentioned here. Early identification of depressive symptoms in high-risk pregnancy is increasingly relevant, particularly in relation to worse maternal and fetal outcomes (27, 38, 49) and for hindering the fullest and healthiest gestational experience possible for each mother-baby dyad and the entire adjacent family nucleus.

However, there are still questions about how to diagnose antenatal depression early. Some recommendations (71, 72) and studies question the implementation of universal depressive symptom screening, mainly using standardized scales to identify symptoms. The lack of more specific and reliable studies justifying universal screening is examined, with the possibility of unnecessary interventions and negative impact on pregnant women, who could become more stressed by the screening procedures. However, most scholars on the subject (73) argue that health professionals and prenatal services, in general, would be adequately qualified to identify depression symptoms and, based on this, carry out the necessary treatments and referrals. Corroborating this opinion, several studies (74, 75) showed that psychological screening can be an effective strategy for mental health care during pregnancy to help identify initial symptoms and for reaching women who, for socio-cultural, emotional, and even medical reasons, would have difficulties identifying symptoms, attributing them to the pregnancy.

Some limitations of this study included the lack of exploration of other risk factors identified in the literature associated with depression during pregnancy, such as assessing the stress perceived by the patient, as well as stressful life events, specific family conflicts, or even domestic violence by a partner. In terms of the participants’ health aspects, a limitation was the lack of information regarding current medication treatment related to existing illnesses, which would add relevant variables to the discussion. Furthermore, in this study, the interview to assess depression in pregnant women took place at a single stage of pregnancy, hindering the identification of whether the disease improved or worsened and how many patients underwent some psychotherapeutic or psychiatric intervention before giving birth. There is no consensus regarding the gestational trimester in which depression would be most prevalent; however, early and late pregnancy is reported as the most susceptible (76), although some studies (77, 78) indicate that this prevalence tends to increase as the pregnancy advances. Longitudinal studies are needed to answer this question, especially in high-risk pregnant women, with additional investigation indicating the cause-effect direction.

The present study reported a high rate of antenatal depression, with approximately one case in every five pregnant women, without difference between low- and high-risk pregnant women, possibly because many low-risk pregnant women were hospital employees with high workload stress levels. Although similar studies corroborate the data, such results highlight the importance of the topic, confirming depression during pregnancy as one of the leading clinical complications during the gestational period in comparison with data on the Brazilian population (79). Other important aspects of this study are the significant sample size and face-to-face interviews with trained psychologists, who administered a diagnostic instrument based on the DSM III (40) when most studies (90%) use a screening scale and self-report to identify depressive symptoms.

However, despite the relevant rates presented here, few physicians notice the presence of depression during prenatal care, and even fewer initiate appropriate treatment (80). Therefore, it is often underdiagnosed, severely affecting maternal or fetal health. Health professionals still make errors and have deficiencies in recognizing, diagnosing, and treating depression during pregnancy, consequently impacting the pregnant woman and newborn. Unfortunately, depression during pregnancy is a common and underdiagnosed condition because its symptoms are often attributed to the pregnancy, and the mental health support necessary is still poorly understood.

Thus, this study brings new information to help recognize the disease and establish early treatment. First, it reinforces the notion that unfavorable economic conditions and the lack of a partner are important risk factors. Moreover, high-risk pregnancy may not be a preponderant risk factor, but some diseases, such as epilepsy during pregnancy, may be an additional risk factor. In all cases, further research is needed on the impact of prenatal care on pregnant women with previous and intercurrent illnesses.

## Data availability statement

The raw data supporting the conclusions of this article will be made available by the authors, without undue reservation.

## Ethics statement

The studies involving humans were approved by CAPPESQ - Comissão de Ética para Análise de Projetos de Pesquisa do HCFMUSP HCFMUSP Ethics Committee for Analysis of Research Projects. The studies were conducted in accordance with the local legislation and institutional requirements. The participants provided their written informed consent to participate in this study.

## Author contributions

JA: Writing – original draft, Project administration, Investigation, Conceptualization. GG: Writing – review & editing, Supervision, Project administration, Conceptualization. RF: Writing – review & editing, Resources. MG: Writing – review & editing, Validation, Supervision, Conceptualization.
